# Fighting pathogens in two battlefields: Antimicrobial defenses in the African lungfish

**DOI:** 10.1371/journal.ppat.1011302

**Published:** 2023-04-27

**Authors:** Elisa Casadei, Irene Salinas

**Affiliations:** Center for Evolutionary and Theoretical Immunology Department of Biology, University of New Mexico Albuquerque, New Mexico, United States of America; University of Massachusetts, Worcester, UNITED STATES

Many vertebrates have evolved life histories that allow them to colonize unique ecological niches and survive where others cannot. A great example are lungfishes, sarcopterygian fish that hold a key phylogenetic position as the closest relative to all tetrapods [1–4]. Lungfishes include 3 genera, the Australian lungfish (*Neoceratodus forsteri*), South American lungfish (*Lepidosiren paradoxa*), and the genus, *Protopterus* sp. that includes the 4 African lungfish species (*P*. *annectens*, *P*. *aethiopicus*, *P*. *dolloi*, and *P*. *amphibius*).

## The African lungfish, an example of a vertebrate with extreme physiological adaptations

Both South American and African lungfish have the capacity to undergo estivation or terrestrialization, a process by which they form a mucus cocoon around their body to protect themselves from desiccation. This extreme physiological adaptation allows them to survive annual droughts and lack of food by lowering their metabolism until favorable environmental conditions return. The terrestrialization process has been mostly investigated in African lungfish, which can stay in this dormant, terrestrial form for years and then return to freshwater once rains return. Researchers have studied the African lungfish for decades, fascinated by their dual aquatic and terrestrial life history and the complex physiological adaptations needed to survive in both environments [3]. Now that the Australian lungfish, a non-estivating species, and the African lungfish genomes have been sequenced [4,5], we have a better understanding of the genetic adaptations needed to be a vertebrate capable of life on water and land. First, a very large genome, the largest so far sequenced, and second, a unique genomic architecture, with extremely long genes with the longest introns so far identified in vertebrate genomes and many transposable elements that can regulate gene expression in a variety of environments [4].

## The African lungfish immune system undergoes dramatic changes during terrestrialization

Extreme physiological adaptations are often tied to extraordinary immunological innovations [6–8]. It was long known that the immune system of African lungfish is very different from that of other jawed vertebrates. *Protopterus* sp. have the largest diversity and numbers of granulocytes of all vertebrates [3,9], a type of innate immune cell well known for their potent microbe-killing functions. African lungfish have large depots of granulocytes in their gonads, guts, and kidneys during the free-swimming phase. We recently discovered that African lungfish invest in maintaining these reservoirs because granulocytes become very important during terrestrialization [9]. Specifically, we demonstrated that during the terrestrialization process, these cells migrate en masse via the blood from their reservoirs (the gonads, gut, and kidneys) into the skin, which becomes inflamed in order to form the cocoon that will protect the animal. Granulocytes do not stop at the skin to defend the lungfish body from pathogenic invaders but leave the body and become an integral part of the cocoon structure where they fight pathogens without causing collateral damage in the lungfish body. Granulocytes are known to form extracellular traps, complex DNA structures decorated with dozens of proteins that have antimicrobial functions [10–12]. Among the proteins that are part of the extracellular trap complex are histones, myeloperoxidase (MPO), neutrophil elastase (ELANE), and several antimicrobial peptides (AMPs) [13]. Not surprisingly, terrestrialized lungfish cocoons contain many granulocytes that form extracellular traps, making the cocoon a structure that concentrates bacteria and stops them from penetrating into the lungfish body [9].

## The importance of antimicrobial peptides for lungfish during terrestrialization

From plants to human, AMPs are present in all living organisms. AMPs are small molecules that are generally amphipathic and cationic, and act as a first line of microbial control for metazoans. AMPs display a broad range of activity against different pathogens, and they normally show rapid killing [14–16]. AMPs are constitutively expressed in all animal barrier tissues including the skin. For instance, many amphibians express a diverse array of different AMPs secreted onto the skin surface [17]. In lungfish, our original studies uncovered 4 beta defensin genes named PdDB-1 to -4 identified from *Protopterus dolloi* skin transcriptomes. Constitutive expression of all PdDB genes was detected in the skin of free-swimming lungfish and upon estivation, PdDB expression increased in the skin [9]. Furthermore, we also detected expression of all 4 PdDB genes in the *P*. *dolloi* cocoon, with PdDB-1 and PdDB-2 being the highest expressed. Whether PdDBs are expressed in other lungfish tissues in free-swimming and estivating lungfish remains to be investigated.

**Fig 1:**
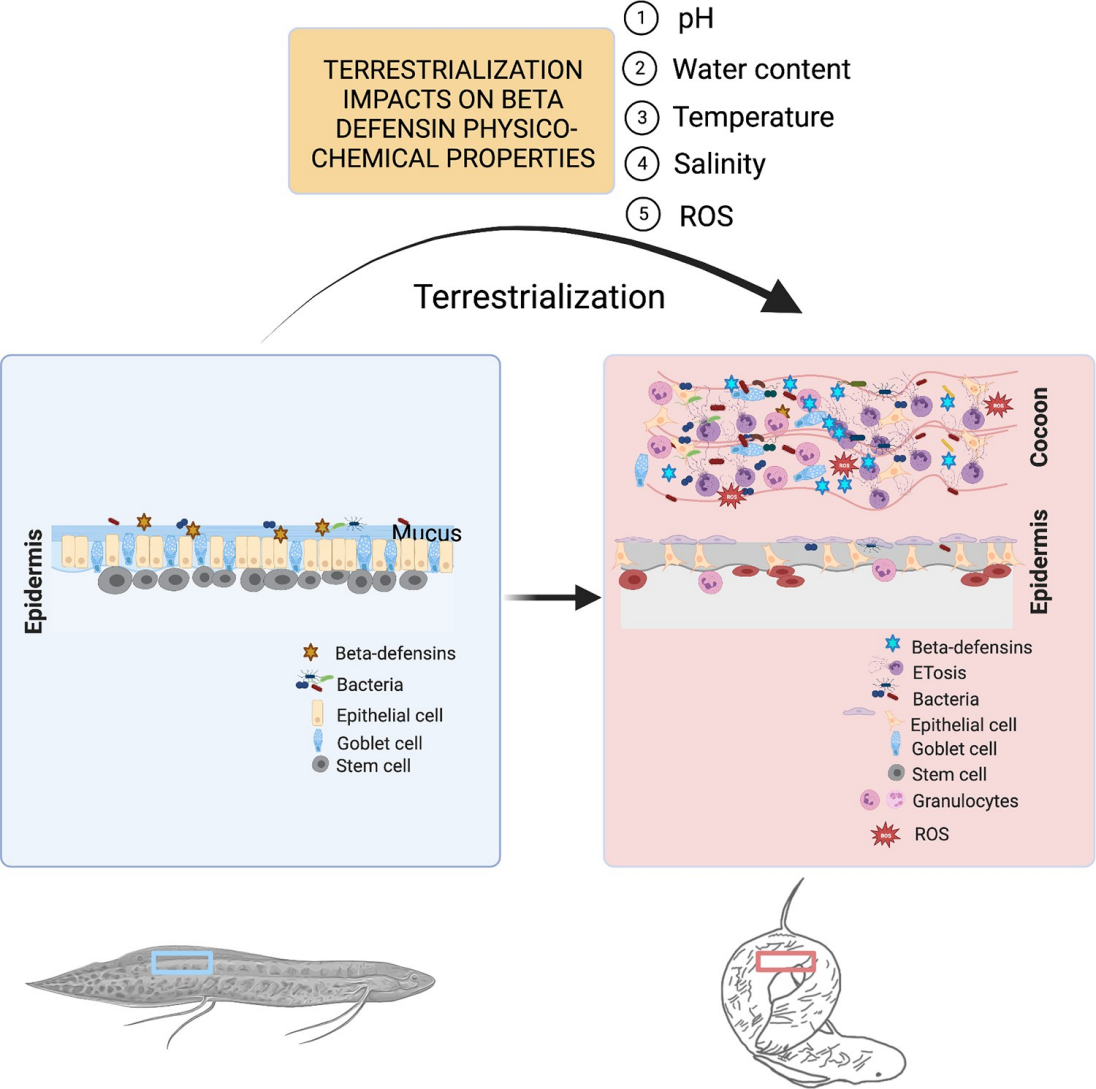
Schematic diagram with the changes that occur in the African lungfish skin upon terrestrialization and the hypothetical environmental factors that may impact AMP structure and function. This figure was made in Biorender.com.

Terrestrialization also drastically changes the environmental conditions that the lungfish skin is exposed. Changes in water content, temperature, salinity, pH, and other environmental factors likely impact AMP structure and function in the terrestrialized lungfish skin and cocoon (Fig 1). Examples in nature have been documented. For instance, oyster AMPs have adapted to high salinity resistance [18] and clavanins (histidine-rich AMPs) have salinity-dependent and pH-dependent biological activities [19]. pH-dependent changes on protein surface electrostatics may not only alter intramolecular bonds and therefore AMP structure but also the way AMPs interact with their membrane targets [20]. Thus, we predict that different beta defensin molecules shift structure and function in freshwater and terrestrialized lungfish skin and that lungfish AMPs have evolved to protect the host against pathogen invasion in both environments. Of particular interest is the cocoon, where we found the presence of many granulocytes undergoing extracellular trap formation [9]. How the physicochemical properties of the cocoon and the external environment impact AMP roles in the extracellular trap complex is yet to be investigated and is an active topic of research.

Perhaps positively charged AMPs in the cocoon may be counterbalanced by the negative charges from reactive oxygen and nitrogen species (ROS and NOS) that are produced in response to pathogens and to the overall oxidative stress generated by the terrestrialization event itself [21,22]. Besides their involvement in the extracellular trap formation, the increased expression of beta defensins in the cocoon may be a response mechanism to dehydration as happens in the amphibian skin in the case of the AMP brevinin-1SY [23]. Since water holes inhabited by lungfish dry out gradually, it is possible that AMP expression in the skin is turned on early during the dry out period, as an anticipatory response before full estivation begins. AMP responses can be driven by the production of ROS and NOS. Such response helps maintain cellular balance and can inhibit neutrophil apoptosis [24]. The production of NOS is likely further supported by the high presence of myeloperoxidase (MPO) produced mainly by granulocytes, an enzyme that converts H_2_O_2_ and chloride to produce hypochlorous acid HOCl, a very reactive, oxidizing agent [25].

## Pending questions and future directions in lungfish immunity

Investigating the immune system of non-model organisms is not an easy task. Immunologists traditionally rely on species-specific reagents such as antibodies, as well as the ability to breed animals in captivity and genetically manipulate these animals. We currently do not have any of those capabilities when it comes to investigating lungfish immunity. However, technologies that are not tied to the species of study, such as high-throughput sequencing, bulk tissue transcriptomes, single-cell transcriptomes, microbiomes, proteomes, and metabolomes combined with the newly sequenced African and Australian lungfish genomes are excellent ways to delve deeper into the antimicrobial arsenal of African lungfish. Unprecedented protein structure predictions using AlphaFold [26] are now available to model 3D structures of any immune molecules of interest. Furthermore, predictions of AMPs from genomes and proteomes are now possible using machine learning applications and curated AMP databases [27–29]. However, given the importance of AMPs and granulocytes for the immunobiology of African lungfish, we still believe that developing specific reagents (i.e., recombinant proteins and antibodies) for some molecules of interest is a worthwhile endeavor that will help resolve specific questions about the immunobiology of this animal.

In summary, the diversity of AMPs in lungfishes is yet to be fully uncovered. The ability of African lungfish to survive in extreme and diverse environmental conditions make them a natural resource of AMPs that likely protect lungfish in both aquatic and terrestrial battlefields.

Lungfish AMPs may harness unique physicochemical and biological functions that could result in novel therapeutics against fish pathogens as well as human pathogens.
